# Regulation of Actin Bundle Mechanics and Structure by Intracellular Environmental Factors

**DOI:** 10.3389/fphy.2021.675885

**Published:** 2021-05-27

**Authors:** Nicholas Castaneda, Jinho Park, Ellen Hyeran Kang

**Affiliations:** 1 NanoScience Technology Center, University of Central Florida, Orlando, FL, United States; 2 Burnett School of Biomedical Sciences, College of Medicine, University of Central Florida, Orlando, FL, United States; 3 Department of Materials Science and Engineering, University of Central Florida, Orlando, FL, United States; 4 Department of Physics, University of Central Florida, Orlando, FL, United States

**Keywords:** actin bundles, macromolecular crowding, cation interactions, actin crosslinker, bending stiffness

## Abstract

The mechanical and structural properties of actin cytoskeleton drive various cellular processes, including structural support of the plasma membrane and cellular motility. Actin monomers assemble into double-stranded helical filaments as well as higher-ordered structures such as bundles and networks. Cells incorporate macromolecular crowding, cation interactions, and actin-crosslinking proteins to regulate the organization of actin bundles. Although the roles of each of these factors in actin bundling have been well-known individually, how combined factors contribute to actin bundle assembly, organization, and mechanics is not fully understood. Here, we describe recent studies that have investigated the mechanisms of how intracellular environmental factors influence actin bundling. This review highlights the effects of macromolecular crowding, cation interactions, and actin-crosslinking proteins on actin bundle organization, structure, and mechanics. Understanding these mechanisms is important in determining *in vivo* actin biophysics and providing insights into cell physiology.

## INTRODUCTION

The dynamic assembly of actin monomers into higher-ordered structures such as bundles and networks is vital to many eukaryotic cell functions. Actin bundle mechanics and structure play essential roles in the formation of filopodia [1, 2], structural support of plasma membrane [3], force generation [4], cell division, and cell motility [2, 5, 6]. Recent studies demonstrate that actin bundles can function as mechanosensors displaying mechanical responses to external stimuli and mechanical deformation [7, 8]. Bundle assembly dynamics are tightly regulated by intracellular environmental factors, contributing to changes in cell mechanics as well as physiology.

**FIGURE 1 ∣:**
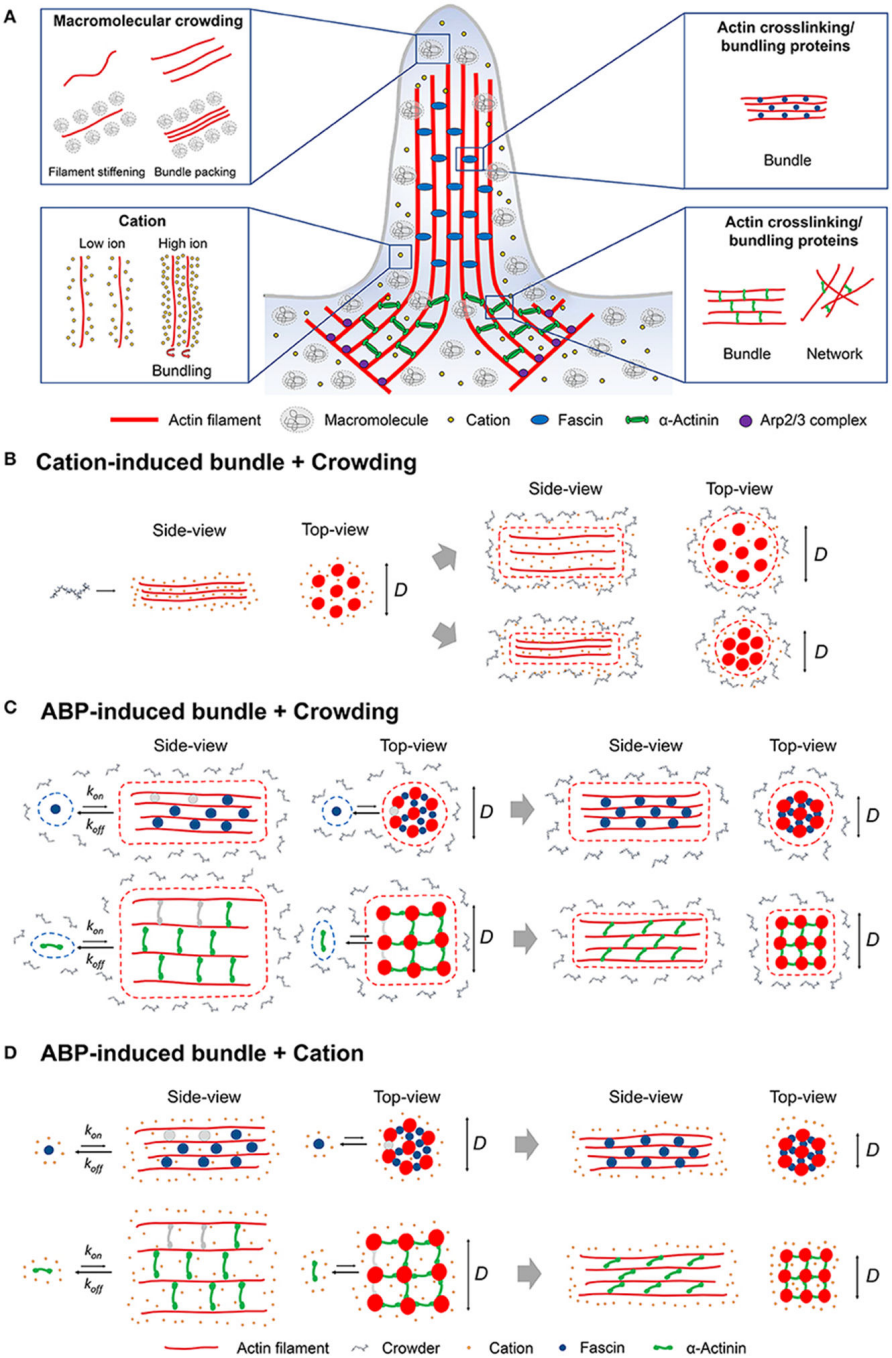
**(A)** Schematic representation of the various intracellular factors such as macromolecular crowding, cation interactions, and actin-crosslinking/bundling proteins (fascin or α-actinin) that can induce actin bundling at the leading edge of a cell. **(B)** Cation-induced actin bundle formation in the presence of macromolecular crowding. Potential interactions between cations and crowding may affect the organization of bundles with a different bundle diameter (*D*). **(C)** Bundles induced by actin-crosslinking proteins [actin-binding proteins (ABPs)] in crowded environments. Potential competitive interactions between ABPs and crowding may affect the binding of ABPs to filaments as well as bundle organization. Actin bundling proteins bind to actin filament (gray; unbound state, blue and green; bound state) during bundle formation with *k*_on_, which is slower than *k*_off_ due to reduced bundling interactions between actin filament and bundling proteins. Compared with small crosslinkers (e.g., fascin; blue), longer crosslinkers (e.g., α-actinin; green) yield a significant decrease in *D* since their orientation switches from perpendicular to angled on filament, affecting bundle compactness [9]. **(D)** ABP-induced bundle formation in the presence of cations can result in modulations to association/dissociation constant *k*_on_ and *k*_off_ by cations influencing bundle organization.

Actin bundle formation can be achieved by macromolecular crowding, electrostatic interactions, and various actin-crosslinking and/or bundling proteins (Figure 1A) [10-17]. Bundles are formed in highly crowded intracellular environments consisting of various macromolecules and ions that limit available cytoplasmic space [18-20]. The presence of macromolecular crowding promotes steric exclusion (“hard”) and/or non-specific (“soft”) effects [21, 22]. Depletion forces induced by macromolecular crowding lead to bundle formation through excluded volume effects, which can overcome repulsive interaction between negatively charged actin filaments [10, 23, 24]. In comparison, cation interactions result in actin bundle formation through counterion condensation [11, 25], similar to DNA condensation [26]. In addition to depletion and electrostatic interactions, cells utilize various actin-crosslinking proteins to form crosslinked bundles or networks [2]. These actin-crosslinking proteins bind actin filaments with different on- and off-rates, influencing the dynamic organization of bundles [15, 27, 28].

The main goal of this review is to summarize major findings on how macromolecular crowding, cation interactions, and actin-crosslinking proteins influence the assembly, organization, and mechanics of actin bundles. In the first part, we describe the effects of depletion and cation interactions on bundle mechanics and structure. In the second part, we introduce the influence of both crowding and cation interactions on the organization and mechanics of bundles crosslinked by actin-binding proteins (ABPs). While the effects of either crowding, cations, or ABPs on actin bundle assembly and mechanics are well-characterized individually, we mainly focus on recent studies demonstrating the potential interplay between these factors on actin-bundling mechanisms.

## EFFECTS OF DEPLETION AND CATION INTERACTIONS ON ACTIN BUNDLE MECHANICS AND STRUCTURE

Macromolecular crowding induces actin bundle assembly by generating depletion interactions [10, 12, 29, 30] through excluded volume effects [31]. Macromolecular crowding promotes attractive interactions between filaments by reducing the free energy required for bundle formation [10, 32, 33]. Depletion forces maximize the overlap between filaments by minimizing the system free energy [33] and generating sliding of filaments [34]. Crowding has been demonstrated to affect actin filament assembly kinetics [35-37], and filament stability has been evidenced by altered critical concentration of actin [38]. A recent study indicates that crowding enhances filament bending stiffness and alters filament conformations, including filament helical twist [39]. Although the effects of crowding on actin filament assembly are known, how crowding modulates bundle assembly kinetics is not well-understood. Changes to bundle assembly by crowding can potentially influence the mechanical properties of actin bundles.

The mechanical properties of depletion-induced actin bundles have been determined by measuring bundle bending stiffness, elastic moduli, and force between filaments (summarized in Table 1) [12, 50]. Previous *in vitro* studies have demonstrated that non-specific depletion forces can function as effective crosslinkers [10, 12, 40]. Bundle bending stiffness depends on the number of filaments per bundle and crosslinker effectiveness demonstrated by mechanical modeling as well as previous experimental evidence [12, 51]. Bundle bending stiffness was shown to quadratically scale with the number of filaments within the bundle [12]. Increasing polyethylene glycol (PEG) concentrations result in enhanced local elastic moduli of actin bundles and networks as well as an increase in bundle diameter, which were determined by microrheological analysis (Table 1) [40]. A recent study using optical tweezers determined the force exerted on two bundling filaments [23] by PEG, resulting in weaker bundling (~0.07 ± 0.006 pN) as compared to divalent cations (Mg^2+^) (~0.20 ± 0.094 pN) (Table 1). Bundles induced by depletion interactions display distinct elastic responses to external forces, such as bending deformations, with minimal evidence of permanent damage [52]. Recently, *in vitro* motility assay and mathematical modeling have demonstrated that depletion-induced bundles exhibit a critical buckling length, which affects the bundle structure and is dependent on the bundle rigidity and the number of filaments in bundles [53]. Martiel et al. [53] demonstrated that as depletion-induced bundles increase in length, they reach a boundary transition that allows for bundle deformations (i.e., loops) although this boundary can be extended depending on bundle stiffness [53]. The relationship displayed between critical buckling length and persistence length as well as the number of filaments in a bundle is a key determinant in bundle deformation [53].

Cation interactions (non-specific electrostatic and/or specific ion binding) promote bundling of actin filaments, which are linear polyelectrolytes, through a reduction in electrostatic repulsion between filaments above a threshold cation concentration required for actin polymerization [17, 25, 54, 55]. High concentrations of divalent cations (e.g., Mg^2+^ and Ca^2+^) were shown to condense actin filaments to bundles and induce over twisting of filaments in bundles, increasing bundle bending persistence length ranging from ~15 to 45μm (Table 1) [16]. Cation binding modulates the mechanics and structure of actin filaments, potentially affecting bundle mechanics and structure. Hocky et al. [56]. demonstrated, through molecular dynamics (MD) simulations that binding of divalent cations at the “stiffness cation site [57]” along an actin filament, induced the reorganization of the DNase-I binding loop (D-loop). Cation binding at the stiffness site generates a tighter twist angle distribution and affects filament torsional stiffness [56]. The addition of counterions further alters the structure of bundled filaments by changing the contact angle per monomer of the filament helices, obtaining an additional twist of ~3.8° [25]. Recently, Gurmessa et al. [41] have shown the effects of varying concentrations of Mg^2+^ on the stiffness and elasticity of bundled networks using optical tweezers microrheology and confocal microscopy imaging. They demonstrated that the stiffness, elasticity, and non-linear force response of the actin network increase with increasing concentration of Mg^2+^ (≥10mM) (Table 1) [41]. Cation binding at discrete sites along actin filaments can lead to bundle formation and promote modulations to bundle structural properties, such as helical twist [16, 25, 41, 58, 59]. Small-angle x-ray scattering (SAXS) showed that actin filaments condensed into bundles display an over twisting of filaments within the bundles [25]. The observed helical twisting of bundles due to cation interactions was corroborated in a recent study that showed cations specifically bind between filaments at key amino acid residues promoting helical twist of bundles [16]. Cation-induced bundles were shown to retain their secondary structures under high pressures (up to ~5 kbar) and temperatures (up to ~60°C), evidenced by Fourier transform infrared (FTIR) spectroscopy [36].

Although investigations into the effects of cations and crowding on actin bundling have been individually shown, the interplay of both factors together has not been well-established. A previous study demonstrated that the onset of bundling promoted by depletion (PEG) and electrostatic interactions exhibits opposite dependence on cation (K^+^) concentrations [29]. A possible competition between electrostatic and depletion interactions can modulate the assembly and organization of actin bundles (Figure 1B). Experimental evidence has demonstrated the individual impacts of both crowding and ionic interactions on actin filament or bundle structure [16, 25]. For example, high divalent cation concentrations condense actin filaments to form bundles, resulting in changes to filament helical symmetry [25]. A potential competition between the bending energy of helical filaments and the binding energy of crosslinkers can contribute to finite bundle sizes [60]. We speculate that the concentrations, types, and size of crowding agents and cations contribute to alterations in actin bundle structure by changes in bending and/or binding energy. A recent study on PEG-induced microtubule bundling indicated that cohesive interactions between microtubules depend on the attractive depletion interactions and electrostatic repulsion [61]. Investigations into the opposite effects of depletion and cation interactions have been performed with DNA [62]. Krotova et al. [62] demonstrated competition, upon an increase in salt concentration between entropy and ionic interactions, of DNA undergoing an unfolding transition in crowded environments. These studies illustrate the counteracting effects of crowding and cation interactions on bundling of cytoskeletal biopolymers as well as DNA condensation. Further studies on the combined effects of both crowding and cation interactions are necessary to determine their impacts on actin bundle mechanics and structures.

## THE INFLUENCE OF CROWDING AND CATION INTERACTIONS ON THE ORGANIZATION AND MECHANICS OF ACTIN BUNDLES CROSSLINKED BY ACTIN-BINDING PROTEINS

Actin-crosslinking and bundling proteins can assemble filaments into higher-ordered structures such as bundles and networks [2, 4, 63]. The size of the crosslinker size, the kinetics of crosslinkers, and the binding affinity of crosslinkers, along with competitive or cooperative interaction between crosslinkers can influence the architecture as well as mechanical properties of actin bundles [15, 27, 28, 42, 51]. The size of actin-crosslinking proteins determines the architecture and compactness of bundles. For example, fascin is an crosslinking protein (diameter ~6 nm), which forms tightly packed bundles, whereas α-actinin is a larger-sized crosslinker (diameter ~35 nm) inducing widely spaced bundles and/or networks [12, 15]. The bending stiffness of fascin- and α-actinin-induced bundles depends on interfilament spacing, supporting an important role of bundle architecture and compactness in bending mechanics of ABP-crosslinked bundles (Table 1) [12, 51]. Binding kinetics (on- and off-rates) and binding affinity of both fascin and α-actinin to filaments have been shown to affect actin bundle assembly and architecture [27, 28]. Competitive interactions between fascin and α-actinin have been shown in a recent study, where fascin-induced bundles, in the presence of α-actinin, were observed to have a reduction in bundle stiffness and filopodia protrusions with varying concentrations of α-actinin [43]. In comparison, combining α-actinin and filamin results in more enhanced elastic moduli of actin filament networks formed by each crosslinker, supporting their cooperative interactions [64].

In a living cell, actin bundles induced by ABPs are formed in a crowded cytoplasm; therefore, it is important to understand how crowding modulates ABP-induced bundling. Changes in filament bending stiffness and conformations in crowded environments [39] can influence interactions between filaments and ABPs, including actin-crosslinking proteins (e.g., fascin and α-actinin) [9] and severing proteins (e.g., gelsolin) [65]. Crowders with different sizes and concentrations [PEG and methylcellulose (MC)] have been shown to affect the organization patterns and potentially nucleation/growth of microtubule bundles crosslinked with microtubule-associated protein (MAP65) [66]. Potential competitive interactions between crowding (PEG, sucrose, and Ficoll) and actin-crosslinking proteins, fascin, and α-actinin, have recently begun to be explored [9]. Macromolecular crowding influences the organization of either fascin or α-actinin bundles by reducing binding interactions between actin filaments and crosslinking proteins (Figure 1C and Table 1), evidenced by fluorescence microscopy and atomic force microscopy imaging along with MD simulations [9]. MD simulations indicated that macromolecular crowding increases interaction energy between fascin or α-actinin and filaments, and reduces the number of hydrogen bonds [9].

Competitive binding of actin-crosslinking proteins, such as fascin and α-actinin, affects their sorting in a size-dependent manner, thereby influencing the actin bundle structure [15]. Furthermore, recent studies suggest that physical confinement has a significant impact on the mechanics and structure of bundles induced by these actin-crosslinking proteins [43, 67]. Giant unilamellar vesicles (GUVs) were used to demonstrate the impacts of enclosed boundary conditions on self-assembly of actin networks and competition between fascin and α-actinin [43]. The physical confinement has a drastic impact on the formation of actin networks, where larger-diameter (>16μm) GUV resulted in a greater probability of actin network/aster formation and reductions in ring formation, directly driving the sorting of fascin [43] and α-actinin [67]. Live cells can introduce a boundary of lipid bilayers that potentially interact with the formation of networks by ABPs [42, 43, 68]. The effects of macromolecular crowding on the self-organization of actin rings by heavy meromyosin (HMM) and α-actinin in confinement have been previously investigated [69]. Crowding agent MC was shown to hinder the contraction of actin rings formed by either α-actinin or HMM in GUVs [69]. Overall, the encapsulation of the actin cytoskeleton can be a regulatory mechanism that facilitates the reorganization of actin bundled networks and potential interactions with lipid membranes.

Cation interactions impact the conformations of actin-crosslinking proteins as well as actin filaments, potentially influencing the mechanics and structure of ABP-induced bundles and/or networks (Figure 1D). The actin filament-binding domain, calponin-homology (CH) domain, is found in various types of actin-crosslinking proteins such as α-actinin and filamin [70]. Divalent cation binding has been shown to induce structural transitioning of the CH domain, impacting actin bundle formation by ABPs. For example, Pinotsis et al. demonstrated that Ca^2+^ binding to α-actinin increases the rigidity of α-actinin, leading to the hindrance of actin bundle formation [71]. Cation binding modulates the bending stiffness of actin filaments [57] and the rheological properties of actin networks [72]. Bidone et al. [72] showed that changes in filament rigidity incurred by specific cation binding result in different strain-stiffening responses of actin networks that depend on the flexibility of actin crosslinkers. Overall, these studies indicate that cation interactions with actin filaments and crosslinking proteins are key modulators in bundle formation as well as mechanics.

## SUMMARY AND OUTLOOK

In this review, we gave an overview of the growing body of work demonstrating how intracellular environmental factors, specifically macromolecular crowding and cation interactions, modulate the assembly, mechanics, and structure of both non-crosslinked actin bundles and ABP-induced bundles. Studies highlighted that both depletion and cation interactions are key players in the tight regulation of actin bundling. Given that actin bundles respond to changes in intracellular environmental factors, it is important to understand (1) whether combined environmental factors act synergistically or competitively to control bundle assembly and (2) how the interactions between actin crosslinkers and crowding and/or cation binding modulate bundle mechanics and structure. Knowledge gained from *in vitro* studies on actin-bundling mechanisms will enhance our understanding of how complex cellular environments influence actin cytoskeleton organization and mechanics. Future studies will benefit from investigating how the actin cytoskeleton actively responds to local changes in intracellular environments as well as external stimuli shaping its architecture, organization, function, and mechanical properties.

## Figures and Tables

**TABLE 1 ∣:** The geometric and mechanical properties of actin bundles induced by crowding, cations, and actin-crosslinking proteins.

Bundle-inducing factor	*D* (nm)	*L* (μm)	*N*	*L*_p_ (μm) or *κ* (pN· μm^2^)	*G*′ (Pa)	*F* (pN)	References
Polyethylene glycol (PEG)	~ 2–20	N/A	~ 6–20	*κ* = ~ 1–10	~ 0.1–1	0.07 ± 0.006	[12, 23, 40]
Mg^2+^	~ 10–350	~ 2–6	~ 4–28	*L*_p_ = ~ 15–45	~ 0.01–0.1	0.2 ± 0.094	[16, 23, 41]
Ca^2+^	~ 10–300	~ 4–5	~ 4–15	*L*_p_ = ~ 12–25	N/A	N/A	[16]
Fascin	~ 10–140	~ 5.4–7.7	~ 3–30	*L*_p_ = ~ 35–170 *κ* = ~ 0.4–10	~ 8.5 ± 0.8	N/A	[13, 14, 42-47]
α-Actinin	~ 94–114	~ 2.0–2.5	~ 3–30	*L*_p_ = ~ 18 *κ* = ~ 0.2–10	~ 44 ± 2	N/A	[42, 48, 49]
Fascin + crowding	~ 108–173	~ 2.3–2.7	~7–13	*L*_p_ = ~ 25–95	N/A	N/A	[9]
α-Actinin + crowding	~ 52–130	~ 3–12	~8–15	*L*_p_ = ~ 10	N/A	N/A	[9]

*D, bundle diameter; L, bundle length; N, No. of filaments per bundle; L_p_, persistence length;* κ, *bending stiffness; G’, Elastic modulus; F, forces between filaments within a bundle*.
